# Procalcitonin Correlates With Cardiovascular Risk Better Than Highly Sensitive C-Reactive Protein in Patients With Type 2 Diabetes in Sub-Saharan Africa: Results From a Cross-Sectional Study

**DOI:** 10.7759/cureus.18357

**Published:** 2021-09-28

**Authors:** Jean-Claude Katte, Andre-Pascal Kengne, Donald Tchapmi, Batakeh B. Agoons, Moffat Nyirenda, Wilfried Mbacham, Eugene Sobngwi

**Affiliations:** 1 Department of Public Health, Faculty of Medicine and Biomedical Sciences, University of Yaoundé 1, Yaoundé, CMR; 2 Department of Medicine, Non-Communicable Diseases Unit, South African Medical Research Council, Cape Town, ZAF; 3 Department of Medicine, Nguelemendouka District Hospital, Ministry of Public Health, Cameroon, Yaounde, CMR; 4 Department of Internal Medicine, Faculty of Medicine and Biomedical Sciences, University of Yaoundé 1, Yaoundé, CMR; 5 Department of Medicine, Non-Communicable Diseases Theme, Medical Research Council/Uganda Virus Research Institute and London School of Hygiene and Tropical Medicine Uganda Research Unit, Entebbe, UGA; 6 Department of Biochemistry, Faculty of Medicine and Biomedical Sciences, University of Yaounde 1, Yaoundé, CMR; 7 Endocrinology and Metabolism, National Obesity Centre and Endocrinology and Metabolic Diseases Unit, Yaoundé Central Hospital, Yaoundé, CMR; 8 Department of Medical Research, Biotechnology Centre, University of Yaoundé 1, Yaoundé, CMR

**Keywords:** africa, inflammation, public health, markers, diabetes

## Abstract

Objective

Inflammatory markers such as C-reactive protein and procalcitonin have been shown to be independent markers of cardiovascular diseases. We aimed to assess the correlation between serum levels of procalcitonin, C-reactive protein and cardiovascular risk in type 2 diabetes.

Methods

We carried out a cross-sectional study at a tertiary level reference hospital in Yaounde, Cameroon. We assessed the cardiovascular risk using the Action in Diabetes and Vascular Disease: Preterax and Diamicron-MR Controlled Evaluation (ADVANCE) cardiovascular risk prediction model in 80 adults with type 2 diabetes. Serum procalcitonin and C-reactive protein were measured in 80 and 76 subjects respectively, using a highly sensitive quantitative enzyme-linked immunosorbent assay (ELISA) method. Correlations were examined using Spearman’s rank correlation test and the correlation coefficients were compared using the Z-test statistic.

Results

Females represented the majority of the study population (62.5%). The median duration of diabetes was 5 (3-10) years and 62.5% of participants had a high cardiovascular risk score. Median serum procalcitonin levels was significantly higher in females compared to male participants: 2.48 (1.76-3.01 ng/mL) vs 1.42 (0.86-1.87 ng/mL); p<0.001. There was no difference in the serum C-reactive protein levels between females and males: 1.20 (0.33-3.33) mg/L vs 0.85 (0.36-2.77) mg/L; p=0.669. Procalcitonin was moderately correlated with cardiovascular risk (r=0.58, p<0.001). The correlation was slightly higher in females (R=0.56, p<0.001) versus males (R=0.49, p=0.005) although not significantly different (Z-statistic=0.734, p=0.463). Serum C-reactive protein did not show a meaningful correlation with cardiovascular risk (R=0.23, p=0.050). At a threshold of 2 ng/ml, serum procalcitonin identified participants with a high cardiovascular risk score, with a sensitivity and specificity of 64% and 80% respectively.

Conclusion

Compared to C-reactive protein, procalcitonin may be a better surrogate marker for cardiovascular risk prediction in this population with type 2 diabetes.

## Introduction

Type 2 diabetes (T2D) is a metabolic disorder characterized by chronic hyperglycemia secondary to insulinoresistance and secondary insulinopenia. It is associated with a two to four-fold increased cardiovascular morbidity and mortality making this a public health challenge [1,2]. Models developed to quantify the future risk of cardiovascular events are commonly used in clinical practice in order to improve the early detection and management of those at increased risk [3,4]. Examples of some commonly clinically relevant prediction models include the Framingham, QRISK, SCORE (Systematic COronary Risk Evaluation) and ADVANCE (Action in Diabetes and Vascular Disease: Preterax and Diamicron-MR Controlled Evaluation) cardiovascular risk scores [5]. These models provide an objective basis for risk stratification by combining multiple predictors in a mathematical formula to estimate the risk of developing a cardiovascular outcome. They however require multiple clinical and laboratory variables that may be difficult to collect in a timely manner in resource-depleted areas. There is therefore a push to identify simpler methods of cardiovascular risk stratification and the use of inflammatory markers in predicting future cardiovascular events is increasingly becoming standard practice.

Commonly studied inflammatory markers and their potential use in cardiovascular risk prediction include interleukin (IL)-6 and the C-reactive protein (CRP). CRP is an acute-phase inflammatory protein that is primarily synthesized by liver hepatocytes in response to infection, trauma and inflammation [4,6]. Serum CRP plays a role in atherosclerosis by directly binding highly atherogenic oxidized low-density lipoprotein cholesterol (LDL-C) and is present within lipid-laden plaques. Elevated levels of serum CRP have been shown to be an independent predictor of cardiovascular disease in individuals with cardiometabolic disorders such as diabetes [7] and even in asymptomatic individuals [8] and has also been used to predict the long-term risk of myocardial infarction, stroke, peripheral arterial disease and sudden cardiac death in apparently healthy subjects [4,6,9]. For this reason, the American Heart Association recommends that patients at intermediate or high risk of coronary heart disease may benefit from the measurement of serum high-sensitivity CRP (hsCRP) [9]. Another recently studied marker and its relationship with cardiovascular risk is the procalcitoinin (PCT).

PCT is a novel marker which is mainly synthesized and released by the follicular cells of the thyroid gland in response to infection or inflammation. It has been shown to be elevated in patients with atherosclerosis [10]. Human macrophage-activated adipocytes are able to secrete PCT which is associated with advanced atherosclerosis, obesity and insulin resistance [11]. One large population-based prospective study demonstrated a positive association between serum PCT levels and cardiovascular risk in subjects with no previous history of acute cardiovascular events [12].

Whether serum PCT correlates with cardiovascular risk and how it compares to other markers such as serum CRP especially in patients with diabetes has not previously been demonstrated. This may potentially be important in rapid cardiovascular risk stratification with implications for the optimization of clinical care. This study, therefore, aims to assess the correlation between serum PCT, CRP and cardiovascular risk in adults with type 2 diabetes. We also sought to determine if serum PCT correlated better than CRP with cardiovascular risk in these patients with type 2 diabetes.

## Materials and methods

Patient and public involvement

Clinicians in the sub-Saharan African region are faced with a common recurring theme for patient concern: the subject of prognosis in people with diabetes. Already validated models such as the Framingham risk depend on more than one laboratory measurement, and therefore add to the already high economic burden of this subgroup. In a bid to reduce cost, yet provide an accurate estimate of cardiovascular risk, we sought to study a single marker and develop a cost-effective basis for cardiovascular risk prediction in people with type 2 diabetes in sub-Saharan Africa. Prior to study design conception, patients (during their routine consultation) were asked if the idea of a single marker was appealing contrary to the multiple laboratory tests to guide their cardiovascular risk assessment. All participants involved in the study received individual results slips for PCT and hsCRP followed by a short discussion to explain the meaning of the results.

Study design, setting and population

**Figure 1:**
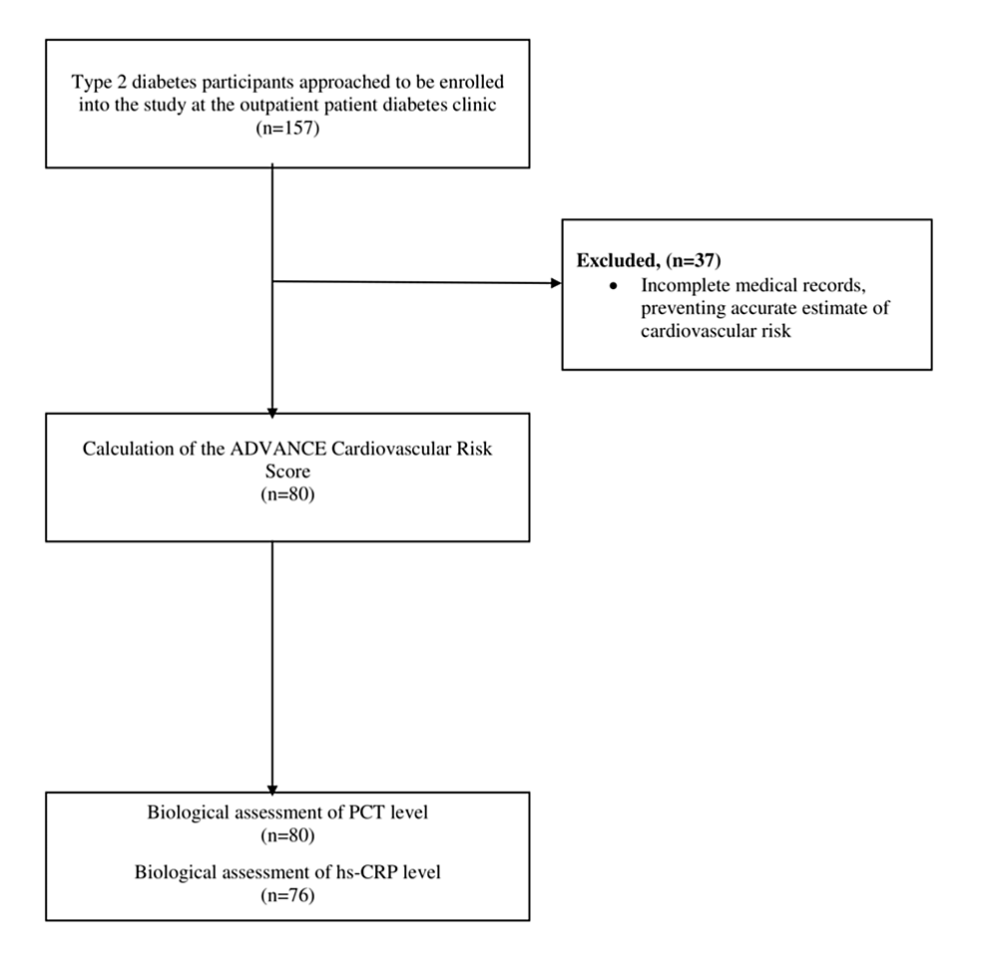
Flow diagram of participant recruitment in the study hs-CRP: high-sensitivity C-reactive protein; PCT: procalcitonin

This was a cross-sectional study carried out at the National Obesity Centre of the Yaounde Central Hospital in Cameroon over a period of 6 months from June to December 2017. This centre was chosen because it is a tertiary centre for the follow-up of people with diabetes in the country. The study population consisted of adult type 2 diabetes patients routinely followed up at the out-patient department of the National Obesity Centre. A list of 157 adult patients with type 2 diabetes who had complete follow-up visits within the last 12 months was obtained from the patient database based on the presence of recent-relevant laboratory results to enable the computing of the cardiovascular risk score. Those with complete medical and laboratory records, who provided written informed consent and who did not have a severe intercurrent disease, clinically recognized infection, overt thyroid disease or pregnancy were retained for the study (Figure 1). This was a preliminary study and therefore did not require sample size calculation.

Prior to subject recruitment, ethical clearance was obtained from the Centre Regional Ethics Committee for Research in Human Health (0696/CRERSHC/2017).

Assessment of cardiovascular risk

Cardiovascular risk was estimated using the ADVANCE cardiovascular risk prediction model which has been described elsewhere [3,13-15]. It involves scoring every participant based on the presence or absence of 10 known traditional risk factors. The attributes retained in the risk prediction model were: age at diagnosis in years, known duration of diabetes in years, gender, presence or absence of atrial fibrillation (ECG done within 1 year), presence or absence of retinopathy (fundoscopy done within 1 year), presence or absence of hypertension and on treatment or not, calculated pulse pressure in mmHg, glycated haemoglobin (HbA1c) (done within 3 months), presence or absence of microalbuminuria (done within 1 year), lipid profile (done within 1 year). The final ADVANCE cardiovascular risk (CVR) score obtained was the estimate of the 10-year cardiovascular risk of developing a major cardiovascular event for each participant in the study. Participants with an ADVANCE CVR score greater than 20 were considered to have a high cardiovascular risk [16]. 

Assessment of procalcitonin and highly sensitive CRP levels

Serum level of PCT was measured using a commercial high-sensitive quantitative sandwich enzyme-linked immunoassay technique (Human PCT ELISA Kit. Catalog No: E-EL-H1492 96 Test) according to the manufacturer’s instructions. The intra-assay and inter-assay coefficients of variation were 10% and 20% respectively. Serum highly sensitive C-reactive protein (hsCRP) was measured by a high-sensitive enzyme-linked immunosorbent assay technique using the nephelometry method on a Genrui Biotech (Shenzen, China) instrument.

Statistical analysis

Data were collected directly into a pre-conceived questionnaire and later recorded into a preformed Microsoft Excel 2010 spreadsheet (Microsoft Corporation, Redmond, USA). Double entry was facilitated by a second data clerk and who also checked item by item for coherency and correctness of the data captured. The final spreadsheet was later exported to Stata SE15 (StataCorp, College Station, USA) for data analysis. Results were presented as frequencies (percentages) or median (first and third quartile) or mean (standard deviation) when appropriate. Chi-square test was used to compare categorical variables across groups while the Mann-Whitney U test was used to compare continuous variables. Correlations were done using the Spearman’s rank correlation test as appropriate. The correlation coefficient values (R values) were transformed into Z scores, which were later compared and analyzed for statistical significance using Z-test statistic. A p-value less than 0.05 was considered statistically significant.

## Results

Baseline characteristics of the study population

A total of 157 participants were initially selected to participate in the study based on the eligibility criteria. Finally, 80 were assessed for the ADVANCE cardiovascular risk score based on available relevant laboratory data. The median (first and third quartile) age of the study population was 59 (50-64) years, with a majority of females (sex ratio of 1.7:1). The median (first and third quartile) duration of diabetes was 5 (3-10) years and the average HbA1c was 7.2 ± 0.7% respectively.

Table 1 shows the baseline characteristics of the study population. Female participants had a significantly higher BMI compared to male participants; 30.2 (27.4-34.4) vs 27.0 (23.9-30.0), p=0.005.

**Table 1:** Baseline clinical and biophysical characteristics of the study population IQR: Interquartile Range, hsCRP: highly sensitive C Reactive Protein, PCT: Procalcitonin, SD: standard deviation, SBP: Systolic Blood Pressure, DPB: Diastolic Blood Pressure, HbA1c: glycated haemoglobin

Participant characteristics	Total	Male	Female	P value
Sample number	80	30	50	
Residence, Urban, n (%)	70 (87.5)	25 (83.3)	45 (90.0)	
Hypertension, Yes, n (%)	38 (48.7)	16 (53.3)	22 (45.8)	
Age, median (IQR), years	59 (50-64)	56 (48-62)	60 (52-65)	0.133
Duration of diabetes, median (IQR), years	5 (3-10)	6 (3-13)	5 (3-9)	0.302
Weight, median (IQR), kg	79.3 (70.1-87.9)	80.1(70.5-87.9)	78.9 (67.9-88.0)	0.941
BMI, median (IQR), kg/m^2^	29.6 (26.3-32.6)	27.0 (23.9-30.0)	30.2 (27.4-34.4)	0.005
Waist circumference, median (IQR), cm	100 (92-105)	98 (89-105)	100 (94-107)	0.178
SBP, mean ± SD, mmHg	132 ± 19	132 ± 21	133 ± 19	0.837
DBP, mean ± SD, mmHg	77 ± 11	79 ± 10	76 ± 11	0.233
HbA1c, mean ± SD, %	7.2 ± 0.7	7.2 ± 0.5	7.1 ± 0.7	0.663
hsCRP, median (IQR), mg/L	1.17 (0.34-3.06)	0.85 (0.36-2.77)	1.20 (0.33-3.33)	0.669
PCT, median (IQR), ng/mL	1.96 (1.341-2.834)	1.42 (0.860 – 1.872)	2.48 (1.765 – 3.015)	<0.001
ADVANCE Cardiovascular risk score	23 (18-24)	21 (17 – 25)	27 (20 – 32)	0.023

Cardiovascular risk estimation of the study population

The median ADVANCE cardiovascular risk score was 23 (18-27). Table 2 shows the characteristics of the study population stratified by cardiovascular risk score. The proportions of patients with low-to-intermediate and high CVR were 37.5% and 62.5% respectively. A higher proportion of female participants 35/50 (70.0%) were at a high CVR compared to male participants. Those with a high CVR were older compared to those with low-to-intermediate CVR; 61 (54-68) vs 55 (48-60), p=0.001. They also had significantly higher circulating levels of PCT compared to their counterparts with low-to-intermediate CVR; 2.485 (1.802-3.045) vs 1.451 (1.014-1.983). BMI, waist circumference, duration of diabetes and hsCRP levels were not significantly different between the two groups.

**Table 2:** Specific biophysical characteristics and biomarker levels stratified by cardiovascular risk IQR: Interquartile Range, BMI: Body Mass Index, SBP: Systolic Blood Pressure, DPB: Diastolic Blood Pressure, hsCRP: highly sensitive C-Reactive Protein, PCT: Procalcitonin, SD: Standard Deviation; PCT: procalcitonin, CVR: cardiovascular risk

Participant characteristics	Low-to-intermediate CVR (0 – 20)	High CVR (>20)	P value
Sample number	N=30	N=50	
Sex, n (%)			
Male	15 (50.0)	15 (30.0)	
Female	15 (50.0)	35 (70.0)	
Age, median (IQR), years	55 (48-60)	61 (54-68)	0.001
BMI, median (IQR), kg/m^2^	27.8 (26.0-30.6)	30.0 (27.4-33.3)	0.099
Waist circumference, median (IQR), cm	97 (89-104)	101 (94-106)	0.053
Duration of diabetes, (IQR), years	5 (3-11)	5.5 (3-10)	0.738
SBP, mean ± SD, mmHg	134 ± 20	129 ± 17	0.277
DBP, mean ± SD, mmHg	76 ± 12	78 ± 9	0.478
hsCRP, median (IQR), mg/L	0.56 (0.26-2.40)	1.27 (0.5-3.51)	0.163
PCT, median (IQR), ng/mL	1.45 (1.01-1.98)	2.48 (1.80-3.04)	<0.001

Highly sensitive CRP and PCT and their association with cardiovascular risk score

Table 2 also shows the circulating levels of hsCRP and PCT based on their CVR score. The median serum hsCRP was 1.17 (0.33-3.06) mg/L and the median serum PCT was 1.96 (1.34-2.83) ng/mL. When stratified by CVR, there was no significant difference in the median serum hsCRP levels although serum hsCRP was higher in participants with a higher CVR score compared to those with a low-to-intermediate CVR score; 1.27 (0.50-3.51 mg/L vs 0.56 (0.26-2.4 mg/L, p=0.163. Contrarily, the median serum PCT levels were significantly higher in participants with a high CVR score compared to those with a low-to-intermediate CVR score; 2.48 (1.76-3.01 ng/mL) vs 1.42 (0.86-1.87 ng/mL); p<0.001.

**Figure 2:**
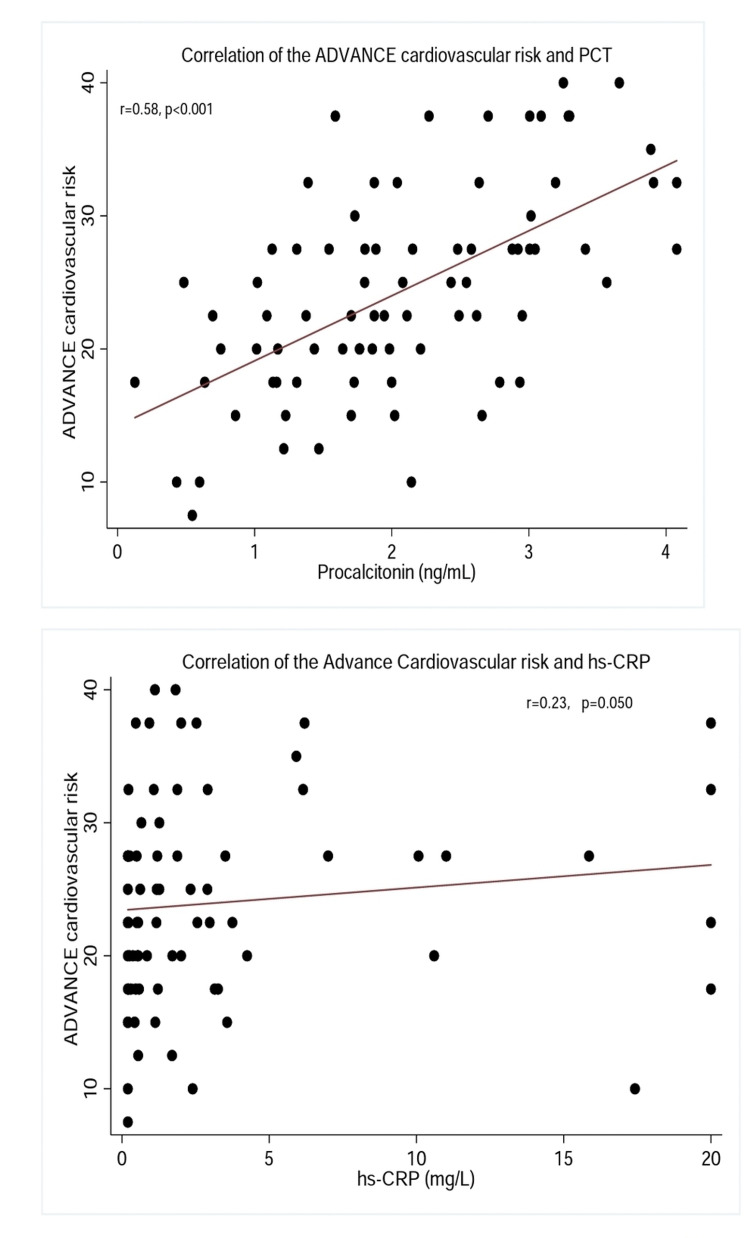
Correlation of the ADVANCE cardiovascular risk with PCT and hsCRP PCT: procalcitonin, hsCRP: highly sensitive C-reactive protein

Overall, serum PCT moderately correlated with CVR (R=0.58, p<0.001), and was not significantly different in females participants (R=0.56, p<0.001) when compared to their male counterparts (R=0.49, p=0.005), after statistical significance assessed by Z-test statistic (Z-statistic = 0.40, p=0.688) shows no difference. There was a generally weak correlation between serum hsCRP and CVR (R=0.23, p=0.050) with the correlation coefficient not different in females (0.28, p=0.051) and males (R=0.11, p=0.602), as shown by the Z-test statistic (Z-statistic = 0.734, p=0.463). Figure 2 shows the scatter plots between the ADVANCE CVR score and serum PCT and hsCRP respectively.

**Figure 3:**
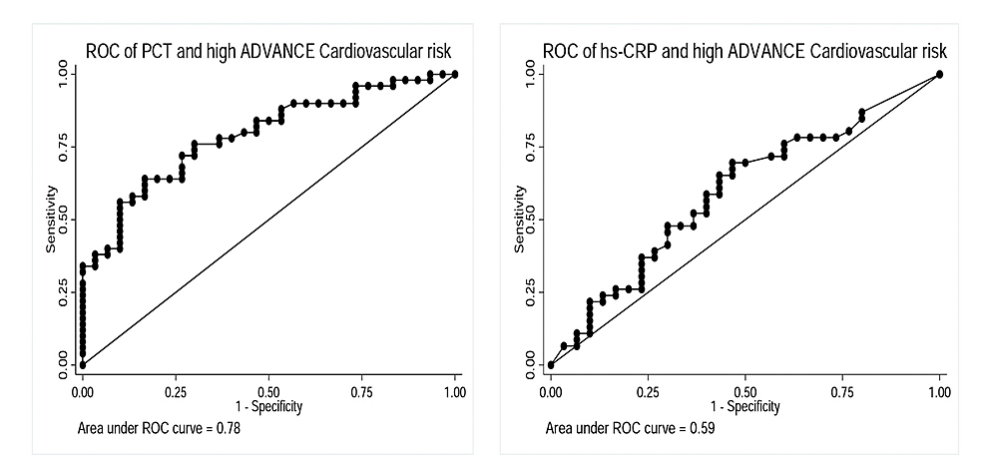
ROC of PCT and hsCRP in detecting high ADVANCE cardiovascular risk ROC: receiver operating characteristics, PCT: procalcitonin, hsCRP: highly sensitive C-Reactive protein

Figure 3 shows the area under the receiver operating characteristics curves (AUROC) for the relationship between serum PCT, hsCRP and high ADVANCE CVR scores respectively. The AUROC shows that serum PCT detects patients with high cardiovascular risk more accurately than hsCRP. The cut-offs with maximal sensitivity and specificity were 2ng/ml and 3mg/L for serum PCT and hsCRP respectively. At these cut-offs, the sensitivity was 64% and specificity 80% for serum PCT to detect high cardiovascular risk (CVR >20). Equivalents values for serum hsCRP were 26.1% and 76.7%. The AUROC for CRP and high cardiovascular risk was 0.59 (95% CI -0.06 - 0.14), p=0.40, and the AUROC for PCT and high cardiovascular risk was 0.78 (95% CI 0.68 - 2.10), p=<0.001.

## Discussion

Results from this study show that serum PCT moderately correlates with cardiovascular risk while serum hsCRP showed no meaningful correlation in this group of patients with type 2 diabetes. Also, those with higher ADVANCE cardiovascular risk scores had a significantly higher level of serum PCT. The findings of this study are similar to the outcomes of multiple studies assessing inflammatory markers and cardiovascular risk, and add to the already existing evidence of the importance of hsCRP, PCT and cardiovascular risk [11,12,17-19]. More importantly, it provides evidence that a single laboratory test using PCT may serve as a more clinically relevant tool in the assessment of cardiovascular risk especially in high-risk groups in a setting where computing traditional cardiovascular risk prediction models may be difficult and expensive.

The serum PCT levels in our study were significantly higher in women, compared to men. This sex difference does not seem to be indicative of a higher level of systemic inflammation in women, as we found no differences with regards to serum hsCRP distribution between the male and female participants. Interestingly, the female participants in this study had a significantly higher BMI, and increased waist circumference compared to the male participants. Increased BMI and waist circumference suggest an increase in adiposity and a greater likelihood of altered lipid profiles and increased pro-inflammatory cytokine secretion. Kozakowski and colleagues found that in postmenopausal women, the abdominal adiposity tends to increase, leading to increased adipocyte secretions of inflammatory cytokines which can induce PCT secretion [20]. The median age of the recruited female participants in our study was 60 years, well past the median expected age of menopause in our setting [21].

The correlation of serum hsCRP and PCT with cardiovascular risk was evaluated according to sex and cardiovascular risk categories. We found significantly increasing levels of serum PCT from low to high cardiovascular risk categories. Serum hsCRP levels were not significantly altered among the different risk categories, implying serum PCT may be better associated with higher cardiovascular risk categories. Also, serum PCT at a given cut-off value had a higher sensitivity and specificity than serum hsCRP in detecting high cardiovascular risk. As such, it can possibly be used to rule in the presence of cardiovascular disease in clinical practice. However, the high cost of the laboratory assessment of serum PCT compared to hsCRP may constitute a limitation to its use, although it remains cheaper when compared to the vast laboratory data that are needed to compute the ADVANCE cardiovascular risk score. The served correlation coefficient between serum PCT and CVR was higher than that observed between serum hsCRP and CVR. Other studies have shown that the use of serum hsCRP as a predictor of cardiovascular disease is potentiated when combined with serum PCT [22].

This study has some limitations to be considered. Firstly, this was a cross-sectional study, and so is subject to the issues of residual confounding; limiting our ability to establish causality between high serum PCT levels and cardiovascular risk. Secondly, we did not have a group of healthy non-diabetic individuals to compare baseline serum PCT and hsCRP levels, although other studies have already established that these inflammatory markers are generally higher in patients with diabetes than in non-diabetic individuals [23-25]. Thirdly, the unbalanced sex ratio might have influenced the observed sex differences in these results. Although changes in estrogen levels have an effect on atherosclerosis and the secretion of cytokines that may influence these inflammatory markers [26,27], the observed pattern might be due to the presence of a higher proportion of women in the study sample which is almost twice that of men. Nevertheless, our findings provide preliminary data to guide future research into the use of these inflammatory markers (PCT and hsCRP) as surrogate estimates of cardiovascular risk in sub-Saharan Africa. Whether interventions geared towards lowering serum PCT levels in patients with type 2 diabetes may decrease cardiovascular risk is a subject for future clinical studies.

## Conclusions

In patients with type 2 diabetes, serum PCT seems to be better correlated with cardiovascular risk than serum highly sensitive C-reactive protein which has previously been shown to be associated with cardiovascular diseases. Serum PCT may therefore be a suitable marker for cardiovascular risk and may serve in risk stratification strategies to predict the development of cardiovascular diseases in patients with type 2 diabetes.
